# An Ordovician variation on Burgess Shale-type biotas

**DOI:** 10.1038/srep09947

**Published:** 2015-04-24

**Authors:** Joseph P. Botting, Lucy A. Muir, Naomi Jordan, Christopher Upton

**Affiliations:** 1 Chatsworth, Spa Road, Llandrindod Wells, Powys, LD1 5EY, UK; 2 Nanjing Institute of Geology and Palaeontology, Nanjing, China; 3 Department of Earth Science and Engineering, South Kensington Campus, Imperial College London, SW7 2AZ; 4 1 Hamilton Terrace, Shoscombe, Bath BA2 8ND, UK

## Abstract

The Cambrian Burgess Shale-type biotas form a globally consistent ecosystem, usually dominated by arthropods. Elements of these communities continued into the Early Ordovician at high latitude, but our understanding of ecological changes during the Great Ordovician Biodiversification Event (GOBE) is currently limited by the paucity of Ordovician exceptionally preserved open-marine faunas. Here we clarify the early stages of the GOBE by describing a new open-marine Konservat-Lagerstätte from the Early Ordovician of Wales. The Afon Gam Biota includes many lineages typical of the Cambrian Burgess Shale-type biotas, but the most abundant groups were sponges, algae and worms, with non-trilobite arthropods being unexpectedly rare. Labile tissues occur abundantly in the sponges and are also present in other groups, including brachiopods and hyoliths. Taphonomic biases are considered and rejected as explanations for arthropod rarity; the preserved biota is considered to be an approximation to the original community composition. We note that other exceptionally preserved communities in the Welsh Ordovician are also sponge-dominated, suggesting a regional change in benthic ecology during the early stages of the GOBE.

Cambrian Burgess Shale-type biotas yield a broadly consistent suite of organisms, with many genera and families shared between continents^1^. By the Middle Ordovician, ecosystems appear to have been much more disparate^2^, with many endemic taxa as well as cosmopolitan ones^3^. However, this transformation has been recognised largely from the record of biomineralised organisms, as there are few Ordovician deposits with diverse soft-bodied fossils^4^. In the Burgess Shale and similar Cambrian faunas, the shelly components are typical of wider communities^5^, and it appears that ecosystems were broadly consistent across wide areas; this cannot be assumed for the more complex Ordovician community development, as most of the few exceptionally preserved faunas currently known yield unique communities^6^,^7^. Although a number of Ordovician Konservat-Lagerstätten have been discovered recently, most are Middle or Late Ordovician in age^7^,^8^,^9^,^10^,^11^, and many represent unusual or marginal environments^6^,^9^,^11^. There are few records of exceptional preservation from the Early Ordovician, and the only diverse open-marine assemblages are those found in the recently-described Tremadocian–Floian Fezouata Biota of Morocco^12^. The Fezouata Biota contains Burgess Shale-type taxa (including many iconic Cambrian groups), but also an Ordovician assemblage (e.g. diverse articulate brachiopods, graptoloid graptolites, and echinoderms including asterozoans and crinoids), plus additional taxa characteristic of later deposits^12^. The sponges are largely represented by families best known from Cambrian faunas^13^. The Fezouata formations were deposited near the Ordovician South Pole, and it is not known whether their communities were typical of Early Ordovician life globally, or represent non-standard communities such as cold-water refugium faunas^14^. In particular, it is unclear whether many Burgess Shale-type taxa survived into the Ordovician in lower latitudes. The diversification history of soft-bodied animals before and within the GOBE is currently uncertain. It is not known whether less-preservable groups underwent rapid diversification in parallel with robustly biomineralising taxa at the start of the GOBE (i.e. beginning in the late Floian^4^), or whether they evolved steadily from the Middle Cambrian onwards^15^. The latter scenario would imply that the GOBE represents the culmination of a long, gradual process rather than being a discrete episode with specific causes and controls. The trace fossil record indicates diversification of some groups throughout the Ordovician^16^, but at least some soft-bodied taxa (e.g. scolecodont-bearing worms^17^) did not diversify significantly before the Middle Ordovician. The Ordovician history of many unmineralised groups is effectively unknown.

In this paper we describe a new exceptionally preserved biota from Wales, UK. The assemblage slightly pre-dates the Fezouata Biota, but is markedly different in ecology: the Afon Gam Biota contains mostly Cambrian-type taxa, but is dominated in abundance and diversity by sponges. Most of the biota would not be preserved without rapid burial of live organisms and/or early soft-tissue mineralisation. The rocks preserving the biota were laid down at a palaeolatitude of approximately 60°S^18^, representing a cool temperate latitude, in contrast to the near-polar Fezouata Biota.

## Results

**Figure 1:**
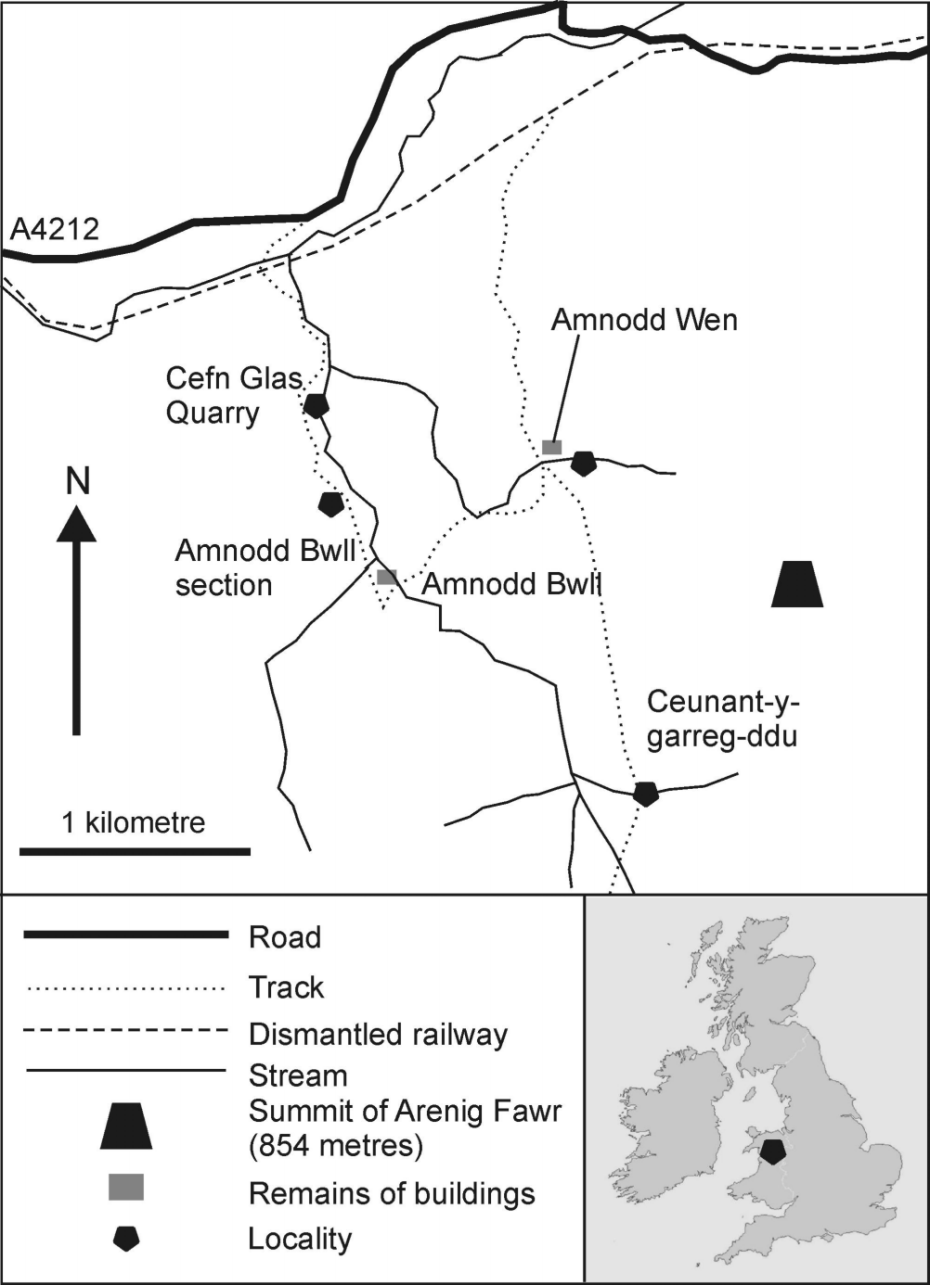
Locality map and stratigraphy of the Afon Gam Biota, North Wales. Image produced using CorelDraw 12.

The biota has been collected from several sections in the Dol-cyn-Afon Formation (early late Tremadocian, *Conophrys* *salopiensis* Trilobite Biozone^19^) around the mountain Arenig Fawr, near Bala, North Wales^20^,^21^ (Fig. 1). The total exposed thickness approaches 200 m, primarily in the long sections of Amnodd Bwll (>70 m) and Ceunant-y-garreg-ddu (>80 m). Within these sections, diverse fossils are distributed intermittently, with the lower 50 m of the Ceunant-y-garreg-ddu section and a 10-m-thick interval in the central part of the Amnodd Bwll section being most productive. In the remainder of these sections, fossils also occur but are more depauperate, limited primarily to indeterminate black streaks, algae and sponges. Additional fossiliferous sections are present in the track-side and stream near Amnodd Wen and in a quarry at Cefn Glas. There appears to be little or no stratigraphic overlap between the sections and localities, and a significant part of the formation remains unexposed in the local area.

The formation crops out widely over a few tens of kilometres, with unusual fossils also having been reported elsewhere from a higher level^22^,^23^. The unit used to be known locally as the Afon Gam Formation, hence the name we have given to the biota. In the surrounding district, coeval sediments are cleaved, distorted, and poorly fossiliferous, but the fossiliferous sections described herein are in virtually undeformed rocks in the strain shadow of a large dolerite intrusion. No exceptionally preserved taxa were found at other locations; the biota therefore appears to be limited to the local area.

**Figure 2:**
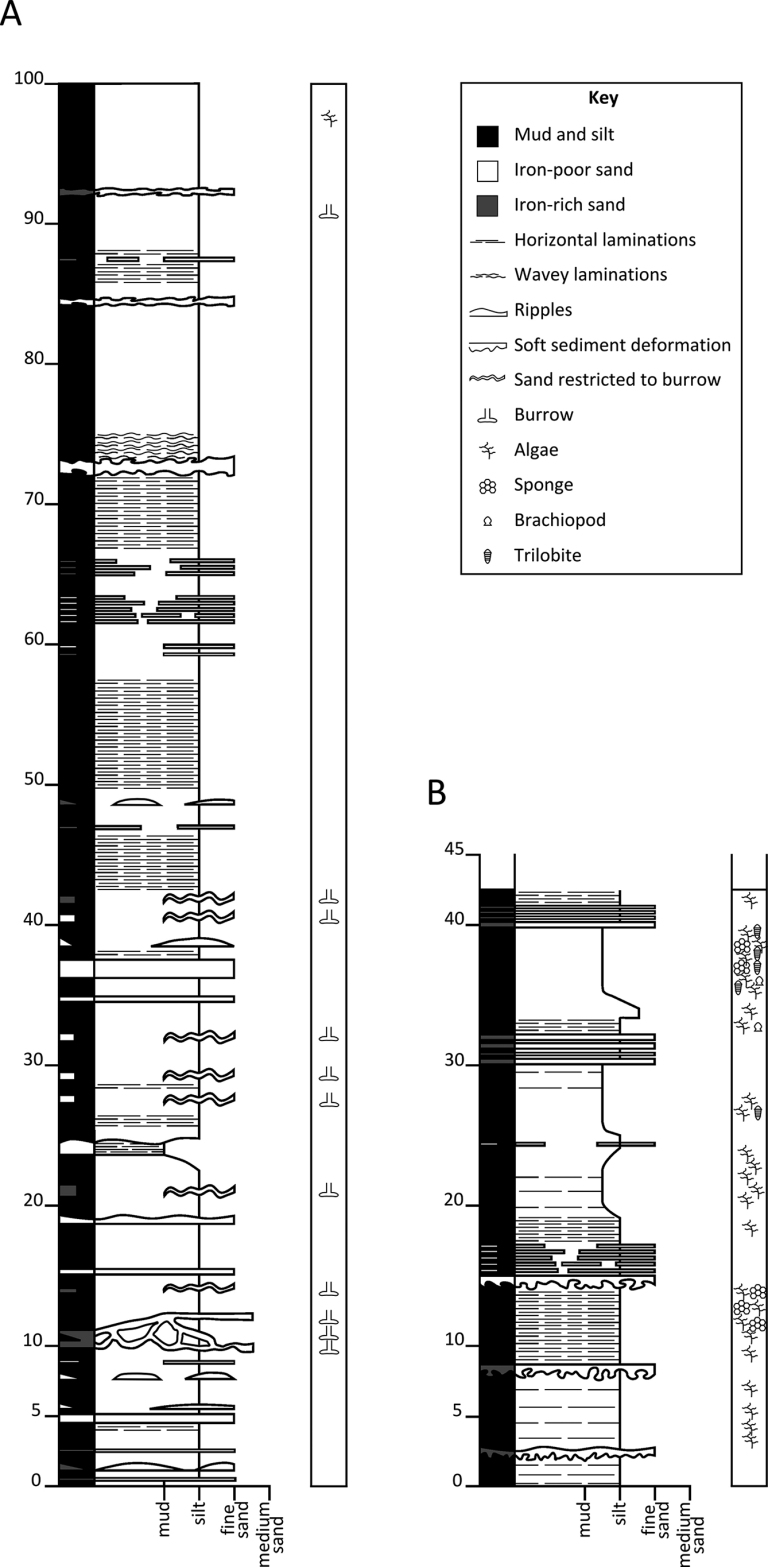
Examples of small-scale sedimentary logs in the quarry at the base of the Ceunant-y-garreg-ddu section. a, log through 1 m of sediment without mass flow deposits, beginning 1 m from base of quarry; b, section through interval 5 m above base of quarry, showing richly fossiliferous mass-flow deposits.

The rock is dominantly composed of siltstone, often significantly burrowed, with sandy laminae and thin mudstone intervals (for example log, see Fig. 2). Much of the sequence was deposited as discrete decimetre-scale beds, each often fining upwards from very fine sand or silt to mud; fossils are concentrated in the middle to upper parts of many beds. Exceptional preservation occurs in non-bioturbated and sometimes also in bioturbated intervals, with burrows excluded from the immediate vicinity of pyritised fossils, presumably due to decay-related chemical conditions. Abundant algae suggest that the biota originated in the photic zone, although it is possible that they represent pelagic rather than benthic species. Damage to the fossils often indicates violent transport and abrupt burial, e.g. fragmented sponges with soft tissues preserved, or rare trilobites preserved in enrolled (defensive) postures. This is interpreted as representing storm- or seismicity-induced offshore transport, with deposition probably occurring around or below storm wave base. Most fossils, including labile tissues, are preserved as iron sulphides (presumed pyrite), with a succession of alteration products on weathering. A hyolith gut is preserved in calcium phosphate, and the phosphatic plates of palaeoscolecidan worms have been replaced by aluminosilicate crystals. Many unmineralised arthropod sclerites are preserved as reflective films, probably composed of aluminosilicates. Skeletal calcium carbonate is normally dissolved, but is occasionally found as recrystallised material in the most robust trilobite exoskeletons, and at some horizons in originally aragonitic shells such as tergomyans.

**Figure 3:**
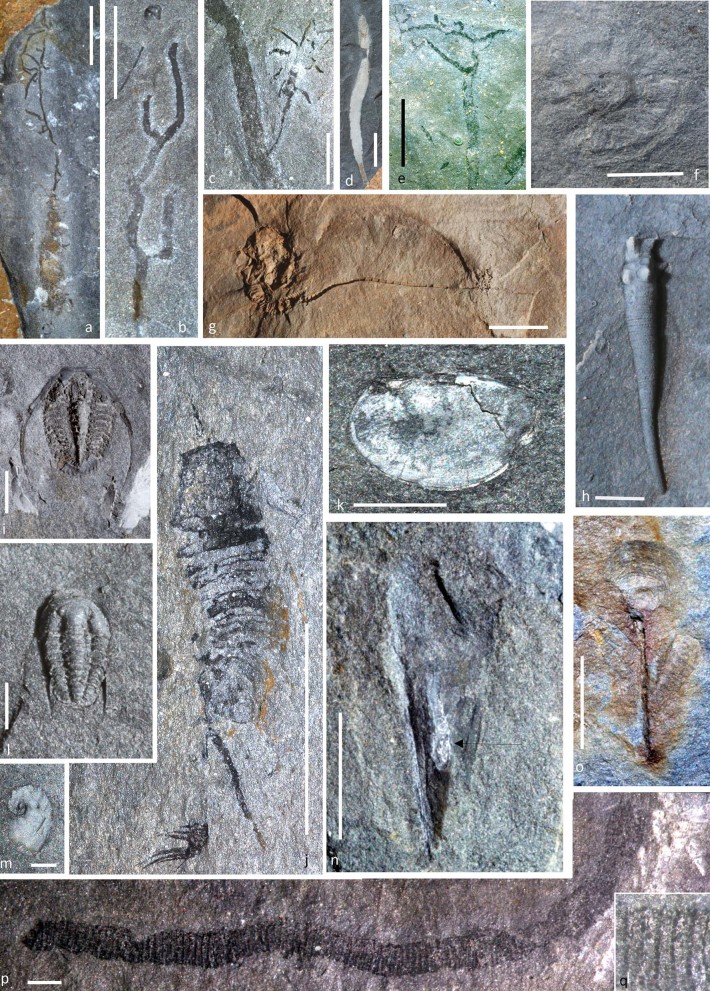
Representative taxa of the Afon Gam Biota, including algae, shelly taxa and arthropods; a, NMW2012.36G.42 large alga with prominent holdfast or flotation structure; b, NMW2012.36G.7, common branching alga; c, NMW2012.36G.88, alga with lateral branch showing probable reproductive structure; d, NMW2012.36G.35, frondose alga; e, NMW2012.36G.54, branching alga with successive thickness reductions; f, NMW2012.36G.91, problematic discoidal fossil; g, NMW2012.36G.44, glyptocystitid rhombiferan *Macrocystella* sp.; h, NMW 2012.36G.91, undescribed problematic echinoderm, whitened latex cast; i, NMW2012.36G.9, remopleuridid trilobite *Pseudokainella* sp.; j, NMW2012.36G.90, undescribed mollisoniid-like arthropod and isolated spinose appendage at lower left; k, NMW2012.36G.22, bivalved arthropod carapace; l, NMW2012.36G.28, trilobite *Shumardia* cf. *pusilla*; m, NMW2012.36G.36, undetermined tergomyan; n, NMW2012.36G.86, hyolith with phosphatised gut (arrowed); o, NMW2012.36G.30, lingulate brachiopod with pyritised pedicle; p, q, NMW2012.36G.85, palaeoscolecidan worm with detail of plate rows (q). Scale bars: a–b, d, f–g: 10 mm; c, e, i–k, n, o: 5 mm; h, l, m, p: 1 mm. Photographs: Joseph P. Botting.

The shelly fauna (Fig. 3g–i, l–o) of the Afon Gam Biota is typical of Tremadocian marine mudstones, comprising mostly trilobites, lingulate brachiopods, tergomyans and hyolithids. Trilobites are the most diverse group, with 15 species known from the formation, including those from previous work^20^,^21^; these species are typical of the shallower parts of the Welsh Basin at this time. The other groups are represented by at least three species each. Shelly fossils occur throughout the studied sections, but are only abundant at particular levels in which discrete event beds are less obvious and soft-tissue preservation is rare. Soft tissues are present in some of these taxa, such as four examples of lingulate brachiopod pedicles (Fig. 3o), a hyolith with preserved gut (Fig. 3n) and another with remnants of other structures. Echinoderms are locally abundant and diverse (around 10 species), and preserved with an unusual degree of articulation. The groups present are stylophorans (including *Anatifopsis*, *Lagynocystis* and cornutes), glyptocystitids, and a problematic, small armless echinoderm (Fig. 3h). The only echinoderm previously reported^20^ from the formation is the glyptocystitid *Macrocystella* (Fig. 3g).

Unmineralised tissue preservation is abundant in algae, sponges and worms, and also present in other groups. Distinct algal fossils include branched (Fig. 3b, e) and strap-like (Fig. 3d) forms. Rare specimens preserve attachment or flotation structures (Fig. 3a), or possible reproductive structures (Fig. 3c). Most algae are fragmentary, but the largest fossils recovered are over ten centimetres long. Among described floras, the fossils perhaps most closely resemble those of the Sinsk Biota^24^ and the Burgess Shale^25^.

**Figure 4:**
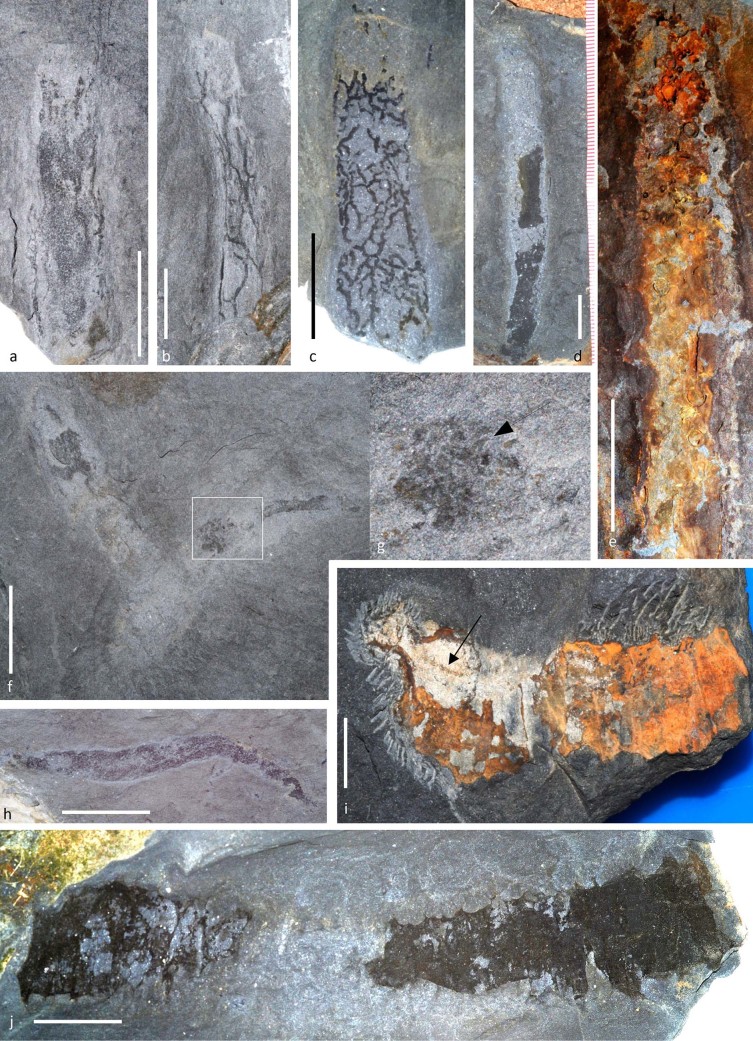
Worms and worm tubes of the Afon Gam Biota. a, NMW2012.36G.40, non-reticulate tube with few thickened strands; b, NMW2012.36G.33, open reticulate tube with longitudinal strand orientation; c, NMW2012.36G.3, finely reticulate tube; d, NMW2012.36G.16, non-reticulate tube in burrow; e, NMW 2013.36G.92, agglutinated tube with echinoderm remains including semi-articulated glyptocystitids; f, NMW2012.36G.87, priapulid worm with proboscis and scalids (g) on collar region; h, NHM AN 15054, example of unidentified vermiform fossil; i, NMW2012.36G.89 *Oikobesalon*-like tube with soft tissues, including central gut (arrowed); j, NHM AN 15055, *Oikobesalon*-like organic tube. Scale bars: 10 mm. Photographs: Joseph P. Botting.

Cnidarians are represented by locally common *Bergaueria*-type trace fossils (presumed anemone resting traces^26^) and perhaps also a much larger, problematic discoidal fossil (Fig. 3f) that appears not to be a jellyfish, but may represent a holdfast. Worm taxa so far recovered are palaeoscolecidans (Fig. 3p, q) and archaeopriapulids (Fig. 4f). There are also numerous possible vermiform remains; an *Ottoia*-like^27^ archaeopriapulid worm preserving detail of the introvert and scalids (Fig. 4f, g) suggests that many indeterminate vermiform fossils with similar preservation (e.g. Fig. 4h) may also be body fossils. Some horizons show mottled bedding indicative of bioturbation by centimetre-wide worms, and there are large numbers of organic burrow linings or tubes that were probably produced by priapulids or their allies (Fig. 4a–d), comparable with those described from the Chengjiang Biota^28^. The diversity of tube structures recovered, ranging from open reticulate (Fig. 4b) to virtually solid (Fig. 4a, d), implies that several species were responsible for their production. Agglutinated tubes constructed primarily from echinoderm remains (Fig. 4e) may have been made by terebellid worms, by analogy with living groups^29^; these are normally found on bedding surfaces, sometimes showing some disarticulation, but some specimens are preserved in oblique section with a sediment-filled interior. An additional tube-like fossil with spinose lateral projections resembles *Oikobesalon*^30^, although one specimen preserves soft tissues, including an apparent central gut (Fig. 4i), and may represent either the trace-maker within the tube or a distinct but superficially similar organism.

Non-trilobite arthropods include a mollisoniid-like species (Fig. 3j), a disarticulating trunk with spinose pygidium, and a possible, poorly-preserved naraoiid. There are also around twenty specimens of well-preserved bivalved carapaces (Fig. 3k). Articulated or semi-articulated unmineralised arthropods are extremely rare; although isolated sclerites are widespread, they are nowhere abundant. An isolated spinose appendage associated with the possible mollisoniid (Fig. 3j) is not assignable with certainty to any group, but somewhat similar structures are present in a range of Cambrian arthropods such as *Sidneyia*^31^.

**Figure 5:**
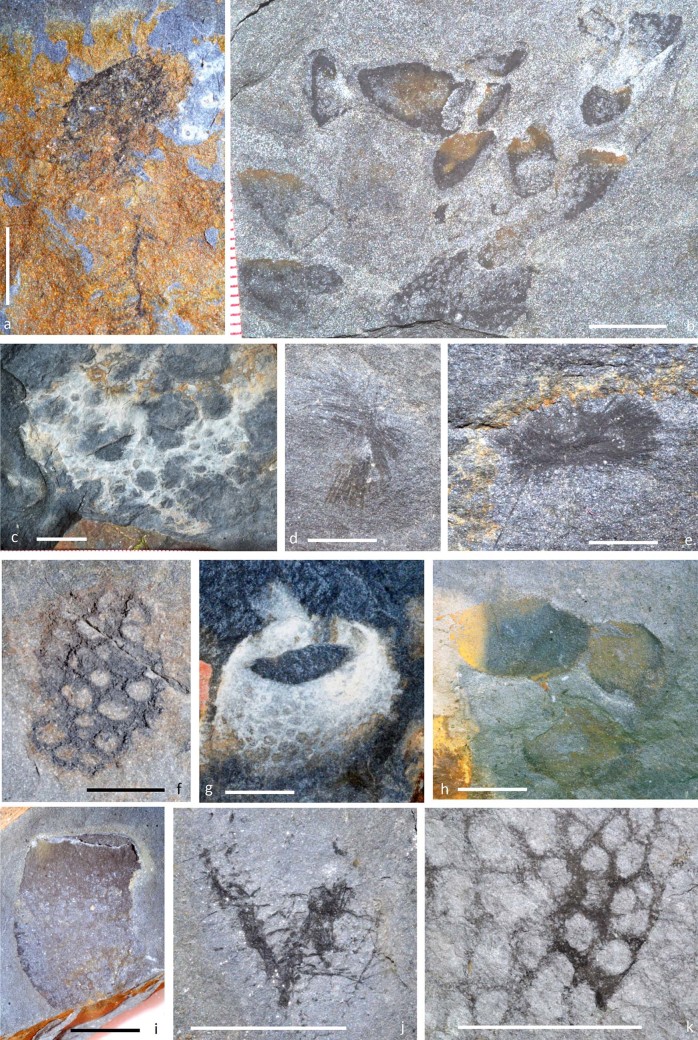
Sponges of the Afon Gam Biota; a, NHM PO 12118, undescribed reticulosan with complex branched stalk; b, NMW2012.36G.19, mass occurrence of undescribed reticulosan; c, NMW2012.36G.17, undescribed large reticulosan with complex array of parietal gaps (holes through body wall); d, NMW2012.36G.4, undescribed *Choia*-like protomonaxonid; e, NMW2012.36G.18, *Choia* sp.; f, NMW2012.36G.14, small reticulosan with pronounced parietal gaps; g, NMW2012.36G.1, *Valospongia bufo*^33^; h, NIGP154640, *Hazelia*? sp.; i, NMW2012.36G.10, *Valospongia* sp.; j, NMW2012.36G.26, *Pirania* sp. (protomonaxonid); k, NMW2012.36G.41, detail of skeleton of *Ratcliffespongia*-like reticulosan. Scale bars: a, d–f, j, k: 5 mm; b, c, g–i: 10 mm. Photographs: Joseph P. Botting.

Sponges (Fig. 5) are the most diverse group, with over 30 species preserved as complete bodies or distinctive partial skeletons; soft tissue outlines are frequently preserved in detail, and most specimens show at least some soft-tissue preservation. The fauna includes a range of protomonaxonid taxa known from Cambrian Burgess Shale-type biotas^32^, such as *Choia* (Fig. 5e), *Hazelia* (Fig. 5h) and *Pirania* (Fig. 5j), numerous reticulosan taxa resembling other Cambrian genera such as *Ratcliffespongia* (Fig. 5k), *Valospongia* (Fig. 5g, i) and *Hintzespongia*^33^, and a suite of other taxa, the affinities of which are uncertain (Fig. 5a–c, f). These additional species include derived sponges without obvious described relatives (Fig. 5a). Many reticulosans show clear parietal gaps in the thin soft tissues (Fig. 5b, c, f, g, i, k).

Of the nearly 100 species currently known from the biota, around two-thirds would not be preserved without rapid burial and/or soft tissue mineralisation. This includes the echinoderms, for which articulated material is required for identification; this normally results from live or very shortly post-mortem burial. The remaining 30–35 species, particularly the trilobites, are typical of Tremadocian faunas in the Welsh Basin^34^.

**Figure 6:**
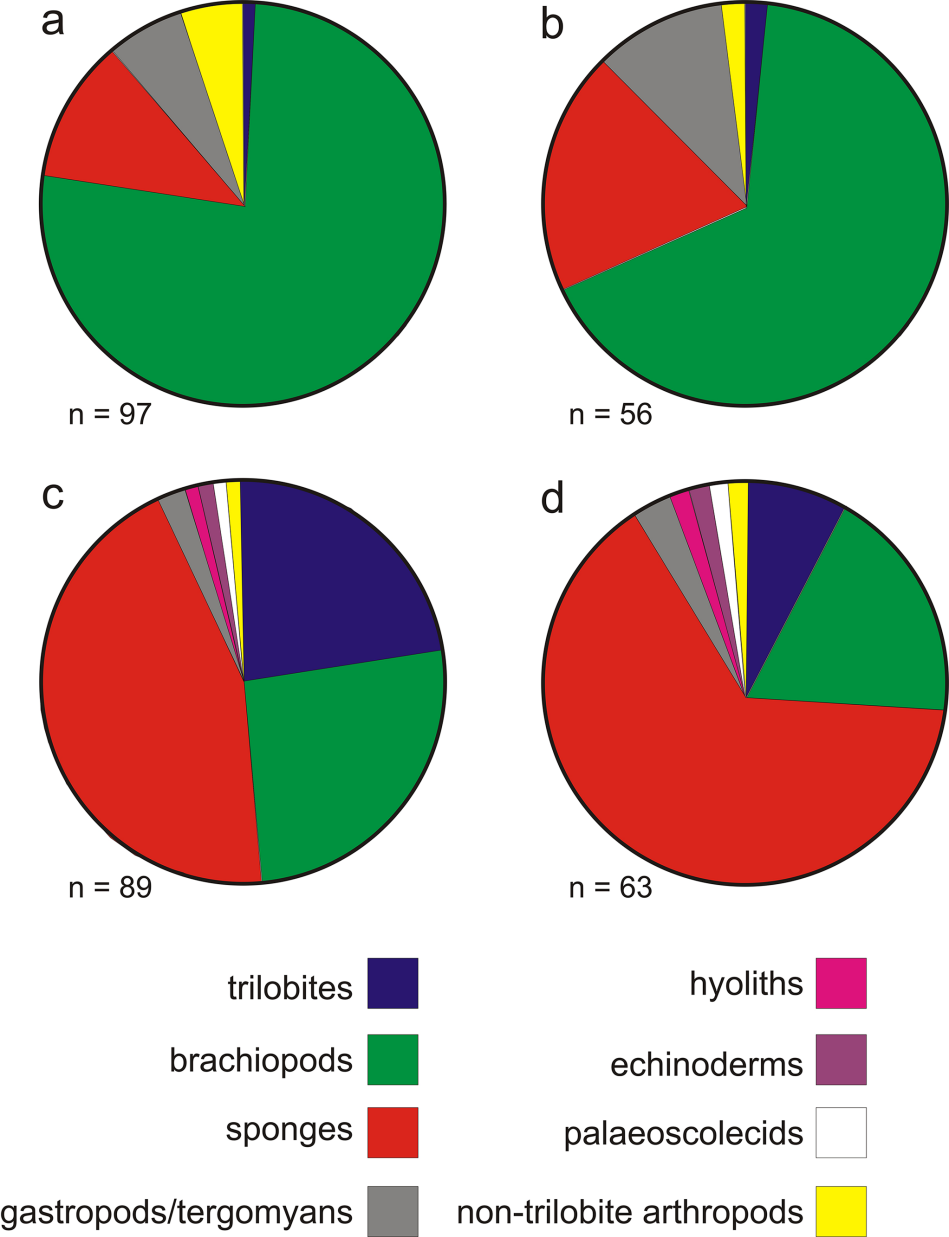
Representative figure showing the relative abundances of major groups (excluding algae and worm traces such as organic burrow linings) within the two most productive systematic samples taken from the Ceunant-y-garreg-ddu section; a, b, Sample CYG2, showing raw specimen counts (a) and modified counts allowing for disarticulation of multi-element skeletons and moulting (b); c, d, the same for Sample CYG7. No attempt has been made to correct the taphonomic over-representation of calcareous and phosphatic taxa in the samples relative to sponges and non-biomineralised groups.

The most abundant elements of the biota in terms of individual fossils are phosphatic brachiopods (Fig. 6), but these are taphonomically concentrated due to the chemical stability of their shells. Excluding these, the most abundant group is sponges (several hundred specimens collected) and algae, followed by worms (represented particularly by unmineralised burrow linings or tubes); however, both algae and worms are difficult to quantify due to fragmentation and ambiguity in identifying imperfect specimens. Presumed algal fragments are more common than sponges, but their relative life abundance cannot be assessed. Tergomyans, trilobites, bivalved arthropod carapaces, hyoliths and some echinoderms (especially *Macrocystella* and a new armless echinoderm) are less abundant, but still moderately common in terms of raw fossil counts; these are likely to be taphonomically over-represented relative to the sponges, algae and worms. Fragments of unmineralised arthropods are uncommon, but in life these creatures may have been more abundant than trilobites. Articulated unmineralised arthropods, most echinoderms, the *Oikobesalon*-like tube and other problematic organisms are known from only a few specimens each, at most, in direct comparison with the abundant articulated sponges.

Although proportions of different groups vary between beds (Fig. 6), the composition of the biota is broadly consistent across all studied localities over a distance of approximately 2 km, with only the rarest groups having been found at only one level. Most species are found across several sites, and at several levels within a site. Some horizons yield relatively high numbers of shelly fossils that are found primarily in those intervals, but this is probably related to taphonomic concentration (see discussion). Sponges often form monospecific assemblages at particular levels, and these must represent a patchy distribution of individual sponge species at a local level.

## Discussion

With any exceptionally preserved biota, among the most critical questions is to what extent the preserved biota accurately reflects the living community. Although there are probably no deposits that preserve all components of the original community (including microbial life and the rarest or largest taxa), some Konservat-Lagerstätten appear to preserve the majority of taxa within the centimetre to decimetre size range. This most notably includes the Burgess Shale^5^,^35^, which has been assessed as preserving all organisms that were present immediately before burial^36^. The community composition of the Burgess Shale is strikingly similar to that seen in the Chengjiang Biota^1^, which is also regarded as preserving most or all of the original community. Most other Cambrian Konservat-Lagerstätten show close similarity to the community composition of these two biotas^1^, or represent a subset of them^37^. This applies particularly to those from open-marine environments, so that there is a general impression that arthropod-dominated faunas represent the standard community type for Cambrian open-marine benthic communities. As later Palaeozoic and modern faunas are also frequently dominated by arthropods, this has led to a widespread assumption that arthropods would have been the dominant component of all normal communities throughout the Phanerozoic^38^. Sponge-dominated early Cambrian faunas are also known^39^,^40^, but these generally do not preserve the soft tissues of the sponges or entirely soft-bodied organisms, and are presumed to represent only a subset of the original community rather than preserving a census of the life assemblage. In contrast, the Afon Gam Biota contains abundant sponges with their soft tissues intact, together with a diverse range of non-biomineralising organisms.

The impression of arthropod dominance in Cambrian deposits is based on the gross composition of the fauna in both diversity and abundance. Individual beds within these deposits can show entirely different dominance patterns, such as high abundance of individual taxa that are normally less common, or dominance by one group of organisms that is normally subsidiary to arthropods, including but not restricted to sponges. If the Afon Gam Biota were restricted to a few isolated beds then this biotic variability in the Cambrian deposits would be a potential explanation of any observed differences from the gross biotic composition; however, the occurrence of broadly homogeneous assemblages throughout the extensive studied sections shows that the observed fauna was neither momentary nor localised.

The Afon Gam Biota appears to illustrate a very different ecological balance from that of the Cambrian Burgess Shale-type biotas. The shelly fauna is typical for the Early Ordovician of the Welsh Basin; the same observation for Cambrian deposits has been used as evidence for the interpretation of the Burgess Shale as a normal benthic community^5^. However, in comparison with the Cambrian faunas, non-trilobite arthropods are extremely rare in our collections, with only a few partially articulated specimens yet recovered. Trilobite fragments are more frequent, but still far less abundant than sponges, especially once multiple moults are taken into account; fully articulated trilobites are rare. Trilobite sclerites were heavily mineralised and therefore could survive long periods of exposure on the sea floor, which would result in complete destruction of sponges and unmineralised organisms. As the taphonomy and sedimentology of the Dol-cyn-Afon Formation indicate repeated mass burial events between periods of slow deposition, with exceptional preservation occurring in numerous discrete horizons, trilobite sclerites would be concentrated during the intervening intervals. This explains the low proportion of complete trilobite exoskeletons recovered.

No trilobite soft tissues have yet been recovered, but this is not surprising. The rarity of fully articulated exoskeletons can be assumed to be due to their relatively low life abundance compared with sponges and worms, while the higher chance of preserving their skeletal remains intact applies in beds both with and without the chemistry needed for pyritisation of labile tissues. The intermittence of soft-tissue preservation through the sampled beds indicates that only a proportion of organisms buried intact should be expected to yield soft tissues; the low numbers of fully articulated trilobites implies that the sample size for this group is not yet large enough. In contrast, non-biomineralised arthropods would only be visible at all when buried under conditions conducive to soft-tissue pyritisation as seen in sponges; they may, therefore, have been somewhat more common than trilobites during life.

Within the numerous horizons preserving soft tissues of sponges and other groups, there is no obvious source of bias against any macroscopic, non- or weakly-biomineralised benthic organisms relative to others. The mechanism of preservation must be presumed to be similar to those seen in other pyritised biotas, and to have been based on microbial sulphate reduction in the presence of dissolved, reduced iron^41^. In Cambrian sponge-dominated communities such as the Niutitang and Hetang biotas^39^,^42^,^43^ the sponges are generally preserved only as articulated arrays of spicules, and soft-bodied organisms are absent from sponge-dominated beds at more productive localities^43^. In the Afon Gam Biota, the sponges are almost invariably preserved with both spicules and soft tissues replaced by pyrite; very rarely is only the spicular skeleton preserved, either articulated or disarticulated, indicating widespread diagenetic loss of spicules in the absence of soft-tissue pyritisation.

If only articulated sponge skeletons had been preserved, without soft tissues, then systematic taphonomic loss of the labile tissues of other groups such as arthropods would have been expected; the abundant soft tissue preservation in sponges implies that such losses cannot be assumed. This is emphasised by the widespread (if in some cases rare) examples of unmineralised worms, algae, brachiopod pedicles and arthropods (including appendages; Fig. 3j) that have been recovered. The abundant organic worm tubes are of a type otherwise recorded only from the Chengjiang Biota^28^, implying that these are not preservable under normal taphonomic conditions and were presumably prone to decay. Preservation of soft tissues by pyrite is also normally associated with faunas that preserve a diverse range of taxa^8^,^44^,^45^ without inherent taxonomic biases, as mineralisation was initiated through the onset of anaerobic decay of soft tissues^46^. It could be argued that in the Afon Gam Biota, mobile organisms were able to escape burial, but this is inconsistent with the observed preservation of worms, and does not explain the rarity of arthropod exuviae or the absence of escape burrows.

Taking into account the sedimentology and taphonomy discussed above, the Afon Gam Biota appears to record a gradation between two fossil assemblage types:

(i) Event beds preserving a fauna dominated by sponges, algae, priapulid(?) worm tubes, with rare examples of arthropods, worms and other groups.

(ii) Intervening intervals, without mass transport but with occasional small-scale burial events, preserving trilobites, agglutinated tubes, echinoderms (locally), algal fragments, and rare examples of other (mostly shelly) taxa.

The two assemblages are not entirely exclusive, especially as concentrations of shelly material on the sea floor may be entrained into event beds. The abundant bioturbation at some levels, particularly in assemblage (ii), indicates the presence of abundant unmineralised organisms that are similar in size to the priapulid tubes and other worms seen in assemblage (i). The remaining question is whether these assemblages represent different sampling of the same benthic community (i.e. different taphofacies), or different life communities.

One explanation of the observed differences is a patchy distribution of different community types on the sea floor, with some areas dominated by sponges and others by a more normal, arthropod- and echinoderm-dominated community. However, this requires that the transported beds with soft tissues, which occur abundantly through the sequence, sampled only the sponge-rich areas. It is possible to envisage a topographic profile with sponges dominating some areas (for example, steep slopes that were prone to slumping), but this leaves unresolved the issue of why the ambient community in the area of deposition was never incorporated into and preserved in any of these beds. It is also possible that there are horizons dominated by non-biomineralised arthropods, which remain to be discovered; if this is the case, though, such horizons must be rare.

Alternatively, the two assemblages can be understood as taphofacies representing the same broad community preserved under different conditions. Assemblage (i) records abrupt burial of an instantaneous community that is dominated by sponges, algae and somewhat rarer worms, but contains brachiopods, occasional examples of trilobites and other arthropods, and other groups. In times between rapid deposition, the more preservable elements of the community were concentrated, and the dominant groups of the life community (sponges, algae, worms) effectively lost. The relatively high number of echinoderms found at some of these levels could result from an environmental preference for more stable substrate conditions, but the concentration of other organisms that would be expected is exactly as seen in assemblage (ii).

There is evidence supporting this taphofacies interpretation in that the local bioturbation indicates a rich unpreserved invertebrate fauna in assemblage (ii), similar to that of assemblage (i) but with greater burrowing density. Additionally, the agglutinated worm tubes composed dominantly of echinoderm remains occur in these more concentrated death assemblages. The scarcity of echinoderm remains outside these tubes could be the result of a sparse life community in which the worms had time to scavenge the majority of echinoderms from the sea floor before burial. Virtually all faunal elements of these trilobite- and echinoderm-dominated units (except for the agglutinated tubes) also occur in the remainder of the sequence within assemblage (i), but in low abundance. The agglutinating organism may have been excluded from those parts of the sequence with high overall sedimentation rate, having required periods of non-deposition that provided sufficient detrital material from which to build or ornament its tube.

Overall, the Afon Gam Biota can be considered to represent a sponge-dominated benthic community. Although there is often an assumption that all early Palaeozoic benthic communities were dominated by arthropods, the taphonomy, sedimentology and preserved community of the Afon Gam Biota cannot easily be reconciled with this view, and although unmineralised arthropods may have been diverse, they appear to have been genuinely scarce relative to sponges and worms. In particular, we cannot present any coherent framework for the biases that would have been required in order to result in the preferential loss of arthropods from the preserved record.

If the Afon Gam Biota does indeed represent a sponge-dominated community with only rare arthropods, then this represents a new variation on the Burgess Shale-type faunas. All the unmineralised taxa found so far are typical of Cambrian faunas rather than later deposits, implying a high level of taxonomic continuity through the late Cambrian and Early Ordovician. There is no evidence in the Afon Gam Biota of groups that are typical of the GOBE (e.g. rhynchonelliform brachiopods, bryozoans, crinoids, lithistid sponges). The entire fauna resembles a reduced version of a Cambrian Burgess Shale-type assemblage, but with a different balance of taxonomic groups. The observed community homogeneity across the area also resembles that seen in the Cambrian deposits, lacking the high levels of ecological segregation seen in later Ordovician communities.

The unexpectedly derived taxa seen in the near-contemporaneous Fezouata Biota, such as xiphosurids and barnacles^12^, are currently unknown in the Afon Gam Biota. Aside from the agglutinated tubes, which are unusual for any Palaeozoic assemblages, and the typical Early Ordovician shelly taxa such as trilobites, only sponges and echinoderms show substantial diversification. The sponge diversity is consistent with a pre-GOBE radiation of sponges in the latest Cambrian and Early Ordovician^47^, and with the timing of an observed diversification of phytoplankton that has been previously suggested as a primary trigger for Ordovician biodiversification^4^. In the Afon Gam Biota there is no replacement of the Burgess Shale-type communities by GOBE-type taxa, even though this had begun in the contemporaneous Fezouata Biota in an apparently similar but higher-latitude environment^12^.

The wider significance of the Afon Gam Biota cannot yet be assessed in detail, but the fauna is suggestive of general changes occurring in the global biosphere. In the Afon Gam Biota, the number of distinguishable sponge species collected over two years equals or exceeds the described totals for all other Cambrian or Ordovician faunas except for the Burgess Shale complex, which has been collected for over a century^32^; in contrast, non-trilobite arthropods are present and possibly diverse, but appear to have been relatively rare. It is possible that the Afon Gam Biota is a unique community type and reflects unusual environmental conditions, even though the shelly fauna is typical of assemblages of this age in the Welsh Basin. However, most other exceptionally preserved faunas known in the Ordovician of Wales are also sponge-dominated with few non-trilobite arthropods^7^,^10^,^48^, suggesting a potential widespread trend.

On a global scale, there are few Ordovician biotas that are directly comparable. In the Fezouata Biota^12^, arthropods are locally abundant and include both Cambrian and post-Cambrian groups. However, there are as yet no ecological data available to assess their importance in the Fezouata community as a whole, and the influence of the polar location on the taxonomic composition also remains unclear. The only other Ordovician offshore Konservat-Lagerstätte is Beecher's Trilobite Bed, which is dominated by trilobites^8^, but may represent a dysoxic environment to which those species were specifically adapted^49^. Most of the remaining exceptionally preserved Ordovician biotas are from marginal or shallow-water settings; in these, arthropods are apparently common but are usually dominated by derived groups such as eurypterids and xiphosurans^9^,^11^,^50^.

The Afon Gam Biota provides a counterpoint to the known assemblages, showing that the development of Ordovician ecology was complex and environment-dependent. For weakly preservable groups other than sponges there was little evidence of large-scale diversification during the Tremadocian of Wales, at least in offshore environments, and the fauna overall was taxonomically continuous with the middle Cambrian biotas. The dominance of sponges suggests that they may have played a much more significant role in Ordovician communities than has previously been recognised, with large areas perhaps resembling some Recent abyssal communities^51^,^52^. The biota also demonstrates how sponge-dominated biotopes can appear in the fossil record as standard trilobitic assemblages under normal sedimentological conditions, which could be severely biasing our view of Ordovician ecological development.

## Methods

The primary localities for the Afon Gam Biota are stream sections at Ceunant-y-garreg-ddu (national grid reference for base of section: SH82103600) and Amnodd Bwll (GR: SH80703683). Additional exceptionally preserved fossils have been recovered from Cefn Glas Quarry (GR: SH80643767) and an overgrown exposure at Amnodd Wen (GR: SH81653747). Searches for similar preservation were conducted without success in the Dol-cyn-Afon Formation at Afon Gam Quarry (GR: SH751422) and Aran Fawddwy (including Pared yr Ychain, centred at GR: SH844227, and outcrops around SH840223), and in the contemporaneous Shineton Shales of Shropshire (including Coundmoor Brook, GR: SJ55530150; Maddock’s Hill Quarry, SJ64500885 and exposures at Nipstone Rock, SO35679648). The material was photographed using a Nikon D80 with Sigma 105 mm macro lens and extension tubes, and photomicrographs were taken using a Canon Eos60D attached to a Leica M125 stereomicroscope. Specimens are deposited in the Nanjing Institute of Geology and Palaeontology (NIGP), the Natural History Museum, London (NHM) and the National Museum of Wales, Cardiff (NMW).

## Author Contributions

All authors conducted extensive fieldwork and contributed to the manuscript, which was written primarily by JPB and LAM with input from NJ and CU; the detailed sedimentology was done by NJ.
